# Psychological Outcomes Following Slow Cortical Potential Neurofeedback in Competitive Golfers: An Exploratory Randomized Trial

**DOI:** 10.3390/neurosci7050109

**Published:** 2026-10-03

**Authors:** Eugenio Lizama, Bastian Carcamo-Herrera, Gonzalo Lopez, Carolina Pereira, Miguel-Angel Serrano

**Affiliations:** 1 Sport Brain Center, 1000 Pembroke Rd, Hallandale Beach, FL 33009, USA; eugenio.lizama@bpgchile.cl; 2 Centro de Investigación para la Resiliencia a Pandemias, Facultad de Ciencias de la Vida e Instituto de Salud Pública, Universidad Andrés Bello, Santiago 8370186, Chile; b.crcamoherrera@uandresbello.edu; 3 Centro de Entrenamiento Neuropsicológico, Avenida Italia 1883, Santiago 7770385, Chile; 4 Department of Psychobiology, Universitat de València, 46010 València, Spain; 5 Behavioral, Affective and Cognitive Neuroscience Laboratory, University of Valencia, 46010 València, Spain

**Keywords:** slow cortical potentials, neurofeedback, golf, self-efficacy, competitive anxiety, attribution, task evaluation, sport psychology

## Abstract

**Background:** Slow Cortical Potential (SCP) neurofeedback trains voluntary regulation of cortical excitability states linked to anticipatory control and prefrontal executive function. In competitive sport contexts, these neural mechanisms may translate into changes in psychological constructs that shape performance under pressure. **Objective:** To explore between-group differences in psychological outcomes assessed during a standardized simulated putting task before and after an eight-week SCP neurofeedback program in golfers, including affective state, self-efficacy and confidence, perceived task workload, and causal attribution patterns, compared with a wait-list control condition. **Methods:** Forty-two active competitive golfers were randomized to a Training group (*n* = 21), completing an eight-week SCP neurofeedback protocol, or a wait-list Control group (*n* = 21). Psychological outcomes were assessed before and after the intervention during standardized competitive settings (simulated putting competitions). Validated instruments included the Self-Assessment Manikin (SAM), the Positive and Negative Affect Schedule (PANAS), self-efficacy and confidence ratings, a Task Evaluation questionnaire, and a sport-adapted causal attribution checklist. Non-parametric statistics (Mann–Whitney U, Wilcoxon signed-rank) were used where normality assumptions were not met, with rank-biserial correlation (RBC) as the effect size measure. **Results:** Affective dimensions (SAM valence, activation, dominance; PANAS negative affect; and STAI state anxiety) remained stable across simulated competitions in both groups. Within the Training group only, state anxiety decreased from pre- to post-task at the second simulated competition (*p* = 0.012), but the corresponding Group × Simulated Competition × Task-phase estimate was not significant (estimate = −1.76, 95% CI [−7.82, 4.30], *p* = 0.560, FDR-adjusted *p* = 0.958), indicating that this within-group change was not differential relative to the Control group, which changed in the same direction. Across the full direct between-group treatment contrasts, none reached statistical significance before or after false-discovery-rate correction (all raw *p* ≥ 0.24; all FDR-adjusted *p* ≥ 0.96). A within-group increase in luck attribution in the Training group (*p* = 0.007) reflected a fully one-directional pattern of change (rank-biserial correlation = 1.00, driven by zero decreases among the nine participants who changed) rather than an unusually large effect. **Conclusions:** These results did not find evidence that this eight-week SCP neurofeedback program produced differential psychological effects relative to a wait-list control. The within-group anxiety, self-efficacy, and luck-attribution patterns reported, before FDR corrections, could serve as hypothesis-generating observations.

## 1. Introduction

Slow Cortical Potential (SCP) neurofeedback is a self-regulation approach that trains individuals to voluntarily modulate slow cortical shifts reflecting changes in cortical excitability and preparatory brain states [1]. Because these slow shifts are closely linked to anticipatory control, excitation–inhibition balance, and the gating of task-relevant processing, SCP neurofeedback provides a plausible mechanistic bridge between trained neural self-regulation and the cognitive–affective processes that shape performance under pressure. In competitive golfers, SCP training has been shown to produce measurable neurophysiological effects consistent with improved regulation-related brain dynamics in ecologically valid competitive settings [2]. Building on that neural validation, the present study tests whether SCP-related self-regulation is accompanied by changes in psychological variables reflecting how athletes perceive control, effort, and outcomes during competition.

Competitive sport constitutes a paradigmatic instance of social stress, a framework that, as Salvador and Costa [3] argued, provides a theoretically richer context for understanding the psychobiological consequences of competition than the classical win–loss outcome paradigm alone. In their integrative model, the competitive situation is preceded by a set of cognitive antecedents—previous experience, social status, personality, expectancies, locus of control, and self-efficacy—that shape the “appraisal” of the upcoming challenge [3]. This primary appraisal determines whether the athlete interprets the competitive demand as a challenge (controllable, effort-dependent) or a threat (uncontrollable, aversive). The resulting coping pattern is fundamentally bifurcated: an active/proactive response—characterized by sympathoadrenomedullary (SAM) activation, increases in testosterone, and positive affect—versus a passive/reactive response—characterized by hypothalamic–pituitary–adrenal (HPA) axis dominance, cortisol rises, and negative affect [3,4]. Critically, post-competition reappraisal, including causal attribution of outcomes, further modulates the psychobiological recovery and conditions future expectancies.

SCP regulation can be interpreted as training anticipatory control over cortical excitability, a process tightly linked to prefrontal executive systems responsible for maintaining task goals, implementing inhibitory control, and stabilizing performance-relevant representations under pressure [5]. In stress-coping terms, these functions are supported by limbic–prefrontal circuitry coordinating appraisal-related control with downstream behavioral and physiological regulation, particularly under uncertainty and evaluative threat [6,7]. Consistent with this perspective, neuroergonomics and neuroimaging work demonstrates that increases in mental workload and performance challenges recruit executive control networks involving lateral prefrontal systems, while regulatory efficiency is reflected in how demand is experienced and managed over time [8,9]. Within the Salvador and Costa [3] framework, prefrontal regulation can be mapped onto the cognitive antecedents phase: the stability of anticipatory cortical states determines whether perceived control, self-efficacy beliefs, and expectancies of success orient the athlete toward the active coping pathway or toward threat-reactive patterns.

In golf, competitive performance unfolds under high evaluative demand and outcome uncertainty. The way athletes appraise situations as challenges versus threats, and the perceived controllability of errors, shapes affective and anxiety responses during execution [10,11,12,13]. These states can compromise attentional control and motor automaticity when pressure rises [14,15]. State affective responses are therefore examined here through brief measures of emotional valence, activation, and control (SAM) and affect (PANAS), which capture the anticipatory and post-competition emotional tone around competition, complementing the broader psychological constructs examined below [16,17]. Importantly, in the Salvador and Costa [3] model, affect is not simply an epiphenomenon of competition but an integral component of the neuroendocrine response pattern: positive affect accompanies active/proactive coping, while negative affect signals the passive/reactive pattern associated with perceived uncontrollability.

Beyond affective state, sustained competitive performance is shaped by psychological constructs occupying key positions in the Salvador and Costa [3] antecedent phase. Self-efficacy—the belief in one’s capability to execute the actions required to achieve desired outcomes—determines whether the pre-competition appraisal resolves toward challenge or threat, and constitutes a central determinant of effort investment, persistence, and stress regulation [18,19,20]. Task Evaluation captures the subjective cost of sustaining cognitive control—a variable that, in the Salvador and Costa framework, maps onto the “effort without distress” factor described by Frankenhaeuser [4]: active coping is associated with high epinephrine and low cortisol, reflecting engagement without aversive load [21]. Finally, causal attribution processes—the post-competition cognitive evaluation of outcomes as internally or externally caused [22,23]—feed back into the reappraisal loop of the model and condition both the recovery phase and future expectancies in subsequent competitive encounters [3,24]. In golf, where small motor and contextual variations produce fluctuating outcomes, the attributional interpretation of results is particularly consequential for sustained confidence and emotional stability.

Mechanistically, SCP neurofeedback can be framed as training anticipatory brain states that reduce uncertainty in performance contexts. Predictive coding accounts propose that the brain minimizes prediction error by updating internal models, with anticipatory control shaping how incoming information is weighted under uncertainty [25,26]. This framework connects naturally with the cognitive activation theory of stress (CATS [27])—referenced in Salvador and Costa [3]—which defines coping in terms of positive response-outcome expectancies: previous success generates expectancies that reduce sustained arousal, whereas repeated failure or uncontrollability generates helplessness or hopelessness patterns. From this integrated perspective, learning to modulate slow cortical shifts may support more stable anticipatory regulation by strengthening the neural substrate of positive expectancy formation, thereby biasing the pre-competition appraisal toward the challenge–proactive coping pole of the Salvador and Costa model. Accordingly, the present study constitutes an exploratory randomized controlled trial examining, for the first time within the social competition–stress framework, whether SCP neurofeedback is associated with pre–post changes in (i) affective state around competition (SAM and PANAS), (ii) self-efficacy and confidence beliefs, (iii) perceived workload and task evaluation, and (iv) competitive appraisal and causal attributions. Given the absence of prior research directly linking SCP-related neural self-regulation to these specific psychological constructs within a competitive sport context, no directional predictions are made for individual variables. Rather, it is broadly expected that, if SCP training strengthens anticipatory cortical self-regulation, this may be reflected—partially or selectively—in shifts consistent with the active/proactive coping pathway of the competition–stress model. Findings are intended to provide an empirical and theoretical foundation for confirmatory research with larger samples and active control conditions.

The participants included in the present study were the same 42 golfers previously reported in Lizama et al. [2] and Lizama et al. [28], and the present work should therefore be considered a continuation of that randomized trial, focusing specifically on its psychological outcomes.

## 2. Methods

### 2.1. Participants and Design

Forty-two active amateur competitive golfers aged 22 to 64 years participated in this exploratory randomized controlled trial (mean age = 43.29 years, SD = 13.02). All participants were active players from golf clubs in Santiago, Chile, and trained at least once per week. The sample covered a wide range of competitive levels, with handicaps ranging from professional and low negative values (−3, −1) to high handicaps of up to 35, ensuring representation of elite, advanced, and amateur golfers. Participants were assigned to a Training group (*n* = 21) or a wait-list Control group (*n* = 21) using stratified randomization based on handicap to balance competitive level across groups. The allocation sequence was computer-generated with Randomizer.org by a team member not involved in participant assessment or contact and remained concealed until each participant had completed baseline procedures; participants were enrolled by a research assistant blind to the sequence, and outcome assessors remained blinded to group assignment throughout, while participants themselves could not be blinded because the Control group was a wait list [2]. The Control group had a mean age of 46.00 years (SD = 12.81) and a mean handicap of 9.71 (SD = 7.88), whereas the Training group had a mean age of 40.57 years (SD = 13.18) and a mean handicap of 7.91 (SD = 9.57). No significant between-group differences were observed in age (*p* = 0.16, Cohen’s d = 0.44) or handicap (*p* = 0.51, Cohen’s d = 0.22). Inclusion criteria comprised an active competition license, right-handed dominance, absence of neurological or psychiatric diagnosis, and no prior neurofeedback experience.

A pre–post between-subjects design was employed, with assessments conducted at two simulated competitions (Simulated Competition 1: pre-intervention baseline; Simulated Competition 2: post-intervention). The Training group completed an eight-week SCP neurofeedback program between simulated competitions, while the Control group completed identical pre–post assessment sessions without intervention during the trial period. Psychological questionnaires were administered digitally in standardized assessment conditions immediately before and after each simulated competition. Given the exploratory nature of the study and the absence of prior data on psychological outcomes of SCP training in competitive sport, sample size followed the a priori calculation used for this cohort [2]: G-Power 3.1.9.7, two-tailed independent-samples test, α = 0.05, power = 70%, and an assumed effect of d = 0.8, yielding *n* = 21 per group. Power was deliberately set below the conventional 80% benchmark given the exploratory, pilot nature of the trial (d = 0.8, 1 − β = 0.70, α = 0.05, two-tailed); the study is therefore underpowered for the small-to-moderate effects observed in the direct between-group contrasts reported below. Considering this, non-significant results should not be interpreted as evidence of equivalence or absence of an effect. Findings should accordingly be interpreted as preliminary and hypothesis-generating rather than confirmatory.

The study was conducted in accordance with the Declaration of Helsinki and approved by the Ethics Committee of the University of Valencia (protocol code 2024-PSILOG-3368845; approved on 11 July 2024). All participants provided written informed consent. The study was conducted during 2024 and was conceived as an exploratory, laboratory-based randomized investigation rather than as a clinical trial; therefore, no formal clinical-trial registration was obtained. The psychological assessments and analyses reported here were planned a priori as part of the original study protocol submitted for ethical approval. No participants were lost, withdrew, or were excluded after randomization: all 42 randomized participants completed the experimental protocol and were included in the analyses according to their originally assigned group.

### 2.2. SCP Neurofeedback Protocol

Equipment and electrode configuration. SCP neurofeedback was administered using ProComp hardware and Infiniti software (version 6.9.0.0; Thought Technology Ltd., Montreal, QC, Canada). The active recording electrode was positioned at Cz according to the international 10–20 system. Three additional electrodes were used to monitor vertical and horizontal electrooculographic activity, and a separate electrode served as ground. Sintered silver/silver chloride (Ag/AgCl) electrodes with conductive paste were used to minimize polarization and slow signal drift. The skin was prepared using an abrasive cleaning paste, and electrode impedances were maintained below 5 kΩ.

Familiarization and initial assessment. Before training, participants attended an introductory session to become familiar with the equipment, visual display, and self-regulation tasks. They were instructed to attempt to generate negative SCP shifts, corresponding to the activation task (“focus”), and positive SCP shifts, corresponding to the deactivation task (“calm”). Initial self-regulation ability was assessed using 10 trials without visual feedback, comprising five activation and five deactivation trials.

During familiarization, participants deliberately produced potential artifacts by moving their tongue, clenching their jaw, tensing their shoulder muscles, and frowning while observing the resulting changes in the feedback display. The trainer explained that these movements could alter the recorded signal and should not be used to achieve the feedback target.

Training schedule and session structure. The intervention comprised 16 sessions delivered twice weekly over eight weeks per participant. Each session lasted approximately 60 min, including approximately 20 min for electrode placement and removal and 40 min allocated to training. Sessions comprised four blocks of 40 trials, with two-minute breaks between blocks, yielding 160 trials per session and 2560 scheduled trials per participant. All sessions were conducted at the same golf facility in Santiago, Chile, by a psychologist certified in neurofeedback and biofeedback. The overall scheduling period for the 21 participants in the Training group extended over approximately 20 weeks; this refers to delivery across the cohort rather than the intervention duration for an individual participant. Session adherence was reported as 100%.

Signal processing and visual feedback. Feedback was based on SCP amplitude relative to a passive reference period. The mean amplitude calculated during this period was assigned a value of zero, and subsequent feedback represented deviations from that baseline. The SCP amplitude estimate was updated every 62.5 ms using the average signal amplitude over the preceding 500 ms.

The display used an arrow to represent the direction of the SCP shift. Upward displacement represented a negative shift relative to baseline, whereas downward displacement represented a positive shift relative to baseline. Participants attempted to produce the direction required by the activation or deactivation task. A rectangle at the top of the screen provided an indication of muscle-related signal noise.

Transfer trials and reinforcement. The third block of each session included 10 transfer trials: five activation trials and five deactivation trials. During these trials, the screen remained blank and no continuous visual feedback about SCP amplitude was provided. At the end of each transfer trial, a smiling face was displayed when the trial objective had been achieved. These trials were intended to provide practice in self-regulation without continuous visual guidance.

Monitoring of adverse events. Adverse events were monitored non-systematically. Participants were instructed to report any unexpected or unwanted symptoms experienced during training or following the intervention.

### 2.3. Psychological Measures

#### 2.3.1. State Affective Responses (SAM, PANAS and STAI)

Emotional valence, activation/arousal, and dominance/control were measured using the Self-Assessment Manikin (SAM) framework [16]. The SAM assesses valence, arousal, and dominance through standardized pictorial scales and, because of its non-verbal format, does not require a language-specific adaptation in the same way as verbally administered questionnaires. Positive and negative affect were assessed using the Spanish-language version of the Positive and Negative Affect Schedule (PANAS [17]), validated in a Chilean population by Dufey and Fernández [29]. This adaptation showed adequate internal consistency, with Cronbach’s α values ranging from approximately 0.78 to 0.86 for Positive Affect and from 0.81 to 0.88 for Negative Affect. Both the SAM and PANAS were administered immediately before and after each simulated competition.

State anxiety was assessed using the Spanish-language State subscale of the State-Trait Anxiety Inventory (STAI-State [30]), which evaluates the participant’s current level of anxiety “at this moment.” The STAI-State consists of 20 items rated on a 4-point Likert scale, with higher scores indicating greater state anxiety. The total score was calculated according to the standard scoring procedure, after reversing positively worded items where appropriate. Participants completed the questionnaire at each assessment point to capture transient anxiety responses associated with the experimental/competitive context. The Spanish-language STAI has shown good psychometric properties in Chilean populations; in a large sample from Santiago de Chile, the State Anxiety scale showed high internal consistency (Cronbach’s α = 0.92 [31]).

#### 2.3.2. Self-Efficacy and Confidence

Self-efficacy, confidence, and perceived opponent self-efficacy were assessed before each simulated competition using brief, study-specific single-item numeric ratings, following recommendations for sport-specific efficacy measurement [18,32] and the pre-competition cognitive-appraisal assessment approach of Costa and Salvador [33]. In that paradigm, before competition and after receiving the task instructions, participants rated a single motivation item (“How important is it for you to complete the task successfully?”) on a Likert scale. This example illustrates the timing and single-item assessment approach, rather than an item measuring self-efficacy or confidence. Following this same pre-competition assessment logic, participants in the present study rated their perceived capacity to succeed (self-efficacy), their confidence in performing well, and their perception of their opponent’s self-efficacy, each on a 1–10 continuous scale administered immediately before the simulated competition.

The intended conceptual distinction was that self-efficacy concerned participants’ perceived capability to meet the specific demands of the putting task, whereas confidence concerned their broader subjective assurance about performing well. The two constructs were assessed separately to explore these potentially different aspects of pre-competition appraisal. However, their empirical discriminant validity was not demonstrated in this study, and overlap between the ratings cannot be excluded. These measures were study-specific single-item ratings, not established, validated multi-item scales; the cited references informed their conceptual framing and administration but do not establish their psychometric validity. Internal consistency cannot be assessed for a single-item measure, and neither the reliability nor the discriminant validity of these ratings could be established in the present dataset. Their results should therefore be interpreted as exploratory findings.

#### 2.3.3. Task Evaluation

After competition, participants rated five dimensions of task evaluation—effort, frustration, perceived performance, stress, and perceived difficulty—on a continuous Likert scale, adapting the post-competition task-evaluation instrument of Costa and Salvador [33], who assessed the same five dimensions on a 0–100 scale based on previous work [34,35]. In the present study, these five items were rated on a 1–10 continuous scale to match the response format used for the other post-competition measures. These ratings are analyzed and reported individually, as exploratory measures of subjective post-competition appraisal, rather than as a validated composite workload score.

#### 2.3.4. Causal Attributions

Causal explanations for performance were assessed after competition using a five-item attribution scale adapted from Costa and Salvador [33], itself grounded in Weiner’s Attribution Theory [22]. Participants rated the perceived contribution of five causes to the competition outcome—own capacity/ability, effort, luck, task difficulty, and opponent’s abilities—on a five-point Likert scale (1 = minimal to 5 = maximal importance). Following the classification used by Costa and Salvador [33], two items (own capacity, effort) were treated as internal attributions and three items (luck, task difficulty, opponent’s abilities) as external attributions. Variables were analyzed individually and aggregated as internal versus external attribution indices [36].

#### 2.3.5. Standardized Competitive Exposure

**Figure 1:**
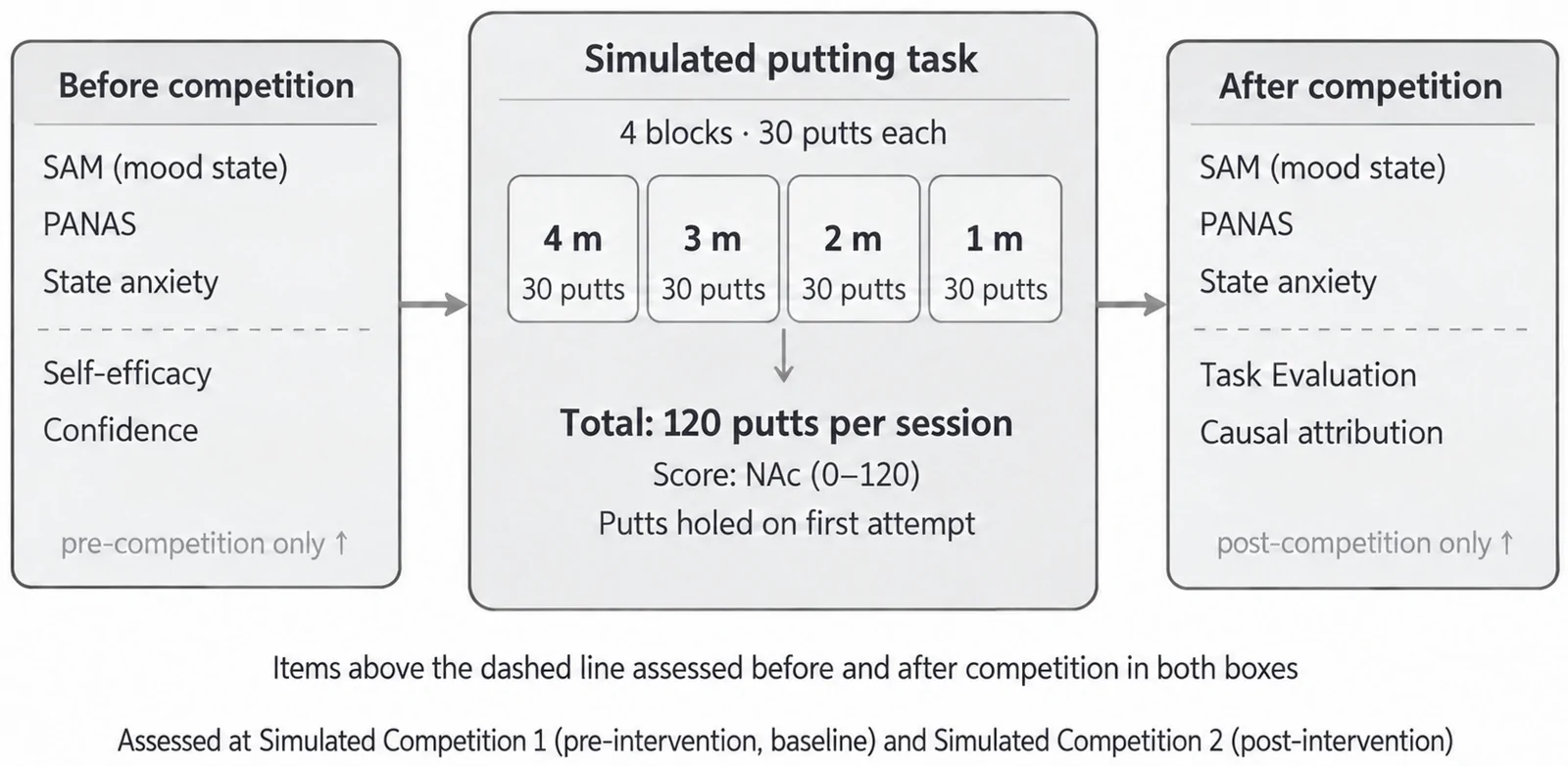
Overview of the simulated putting task and measurement schedule across the competition session.

Sports performance was evaluated using a standardized simulated putting task [37]. Each participant performed 120 putts per assessment, both at baseline and post-intervention, divided into four distance blocks of 30 putts from 4 m, 3 m, 2 m, and 1 m, respectively. The task was carried out on the same natural green, using the same hole and starting points, to ensure stable testing conditions across assessments. Importantly, this procedure did not constitute a formal golf tournament or head-to-head competition. Rather, it was designed to place golfers in a standardized simulated competitive context incorporating time constraints, repeated performance evaluation, standardized scoring, and awareness that their performance was being assessed. These features were intended to reproduce some of the evaluative demands associated with competition while maintaining experimental control. Performance was operationalized as the number of successful putts holed on the first attempt (NAc), yielding a total score ranging from 0 to 120, as well as distance-specific scores for each block. This measure was obtained at baseline and post-intervention to characterize performance under the simulated competitive conditions (see Figure 1). Nevertheless, the task should not be considered equivalent to an actual golf tournament, and its capacity to reproduce the full psychological and ecological demands of real-world competition is necessarily limited.

The putting-performance measure is described here solely to characterize the competitive task and experimental protocol; the corresponding performance analyses and results from this same cohort have been reported separately in Lizama et al. [28] and are therefore not repeated in the present manuscript.

Psychological measures were administered at specific timepoints within each simulated competition session: mood state (SAM), positive and negative affect (PANAS), and state anxiety (STAI) were assessed both immediately before and immediately after the competition; self-efficacy and confidence ratings were assessed before the competition only, as pre-competition cognitive antecedents of the challenge-versus-threat appraisal; and task evaluation and causal attribution ratings were assessed after the competition only, as retrospective evaluations of experienced task demands and outcome interpretation, respectively.

### 2.4. Statistical Analysis

Outcome distributions were examined using the Shapiro–Wilk test and inspected for floor and ceiling effects. Mann–Whitney U tests were used for descriptive between-group comparisons at individual assessment points, and Wilcoxon signed-rank tests were used for descriptive within-group comparisons. Rank-biserial correlations (RBCs) were reported as effect sizes for these non-parametric tests. These analyses were distinguished from the direct between-group change comparisons described below. In particular, a statistically significant change within one group but not the other was not interpreted as evidence of a differential intervention effect.

The principal inferential analyses compared participant-level change scores between the Training and Control groups using Welch’s independent-samples *t*-test, which allows unequal variances between groups. Simulated Competition 1 and Simulated Competition 2 denoted the pre-intervention and post-intervention assessment occasions, respectively; assessment phase denoted the pre-task or post-task measurement within each competition.

For outcomes represented by one score per participant at each competition—self-efficacy, confidence, perceived opponent self-efficacy, task-evaluation items, individual causal-attribution items, and the internal and external attribution indices—individual change scores were calculated as the Competition 2 score minus the Competition 1 score. The direct effect estimate was the mean change in the Training group minus the mean change in the Control group. This estimate represents a Group × Competition change contrast, addressing whether change across the intervention period differed between groups. SAM scores were analyzed using the same procedure, using the specific items and the sum of them.

For PANAS positive affect, PANAS negative affect, and STAI state anxiety, which were assessed before and after the task at both competitions, a within-task change score was first calculated at each competition as post-task minus pre-task. For each participant, the within-task change at Competition 1 was then subtracted from the within-task change at Competition 2. These participant-level double differences were compared between groups using Welch’s *t*-test. The effect estimate was the mean double difference in the Training group minus that in the Control group. This represents a Group × Competition × Assessment-phase contrast and tests whether the change in the within-task response across competitions differed between groups. It does not constitute a general test of all possible intervention-related differences in overall outcome levels.

Each participant contributed one derived change score or double-difference score to each relevant between-group comparison. Repeated observations were therefore combined within participants before the between-group tests were performed, rather than treated as independent observations. The terms Group × Competition and Group × Competition × Assessment-phase describe the contrasts of interest; the reported estimates were obtained through Welch’s tests on these derived scores.

Direct between-group effects were expressed in the original units of each outcome and reported with 95% confidence intervals, raw *p*-values, and Benjamini–Hochberg false-discovery-rate-adjusted *p*-values. The FDR procedure was applied at the 5% level across a single family of [confirm the number of contrasts included in the final correction] direct between-group comparisons. The descriptive Mann–Whitney U and Wilcoxon signed-rank tests were outside this correction family, and their *p*-values were treated as unadjusted exploratory results. The reported 95% confidence intervals were not adjusted for multiplicity and should not be interpreted as simultaneous confidence intervals.

The direct change-score analyses and multiplicity correction were performed in Python 3.14 using SciPy and statsmodels; the descriptive analyses in Table 1, Table 2, Table 3, Table 4 and Table 5 were performed in SPSS version 29.0. All psychological analyses were interpreted as exploratory. Non-significant findings were interpreted as an absence of statistically supported differential change detected in this sample, rather than evidence of equivalence, absence of an effect, or ineffectiveness of SCP neurofeedback.

## 3. Results

Descriptive statistics are presented in Table 1, Table 2, Table 3, Table 4 and Table 5, and the direct Training-versus-Control contrasts are summarized in Table 6. Across the 22 contrasts, no statistically significant between-group difference was detected before or after Benjamini–Hochberg correction (all raw *p* ≥ 0.242; all FDR-adjusted *p* ≥ 0.990).

### 3.1. State Affective Responses

#### 3.1.1. Self-Assessment Manikin (SAM)

Emotional valence, activation, and dominance/control were assessed using the Self-Assessment Manikin (SAM), with descriptive statistics presented in Table 1. The descriptive within-group comparisons between Simulated Competition 1 and Simulated Competition 2 were non-significant for all three dimensions (all *p* ≥ 0.05; RBC range: −0.43 to −0.11). Separate between-group comparisons at each simulated competition were also non-significant. These comparisons do not test differential change between the Training and Control groups.

**Table 1:** Self-Assessment Manikin scores across simulated competitions.

Variable	Simulated Competition	Control M ± SD	Control Mdn [IQR]	Training M ± SD	Training Mdn [IQR]
Valence	1	4.43 ± 0.68	5.00 [1.00]	4.24 ± 0.70	4.00 [1.00]
	2	4.38 ± 0.50	4.00 [1.00]	4.14 ± 0.73	4.00 [1.00]
Activation	1	4.05 ± 0.67	4.00 [0.00]	3.86 ± 0.65	4.00 [1.00]
	2	3.76 ± 0.70	4.00 [1.00]	3.71 ± 0.72	4.00 [1.00]
Dominance/control	1	3.71 ± 0.64	4.00 [1.00]	3.67 ± 0.58	4.00 [1.00]
	2	3.67 ± 0.66	4.00 [1.00]	3.48 ± 0.68	3.00 [1.00]

Note. 1 = Simulated Competition 1, pre-intervention; 2 = Simulated Competition 2, post-intervention; Control = group with no intervention; Training = group with SCP intervention; M = mean; SD = standard deviation; Mdn = median; IQR = interquartile range. Values are presented as mean ± SD and median [IQR].

The direct between-group change contrasts, calculated as the mean change from Simulated Competition 1 to Simulated Competition 2 in the Training group minus the corresponding change in the Control group, provided no statistically significant evidence of differential change in valence (estimate = −0.05, 95% CI [−0.54, 0.44], raw *p* = 0.846, FDR-adjusted *p* = 0.990), activation (estimate = 0.14, 95% CI [−0.42, 0.71], raw *p* = 0.612, FDR-adjusted *p* = 0.990), or dominance/control (estimate = −0.14, 95% CI [−0.59, 0.30], raw *p* = 0.518, FDR-adjusted *p* = 0.990; Table 6).

The arithmetic sum of the three SAM dimensions included in the previous analysis showed no significant within-group change (both *p* ≥ 0.05; RBC range: −0.38 to −0.29) or direct between-group change contrast (estimate = −0.05, 95% CI [−1.03, 0.93], raw *p* = 0.922, FDR-adjusted *p* = 0.990; Table 6).

#### 3.1.2. PANAS and Anxiety

Descriptive statistics for PANAS positive and negative affect and STAI state anxiety are presented in Table 2. For negative affect, comparisons between Simulated Competition 1 and Simulated Competition 2 were non-significant for both pre-task and post-task scores in either group, as were between-group comparisons at each assessment (all *p* ≥ 0.05). For positive affect, the Training group showed a non-significant reduction in pre-task scores between simulated competitions (*p* = 0.079; RBC = −0.448), whereas the corresponding comparison in the Control group was also non-significant. Post-task positive affect showed no significant changes between simulated competitions in either group, and between-group comparisons were non-significant (all *p* ≥ 0.05).

For state anxiety, within-task pre-to-post comparisons were non-significant in both groups at Simulated Competition 1 (Training: Z = −0.872, *p* = 0.383, RBC = −0.234; Control: Z = −1.577, *p* = 0.115, RBC = −0.400). At Simulated Competition 2, state anxiety decreased from pre-task to post-task in the Training group (Z = −2.513, *p* = 0.012, RBC = −0.673), with lower post-task scores in 13 of 21 participants. The corresponding decrease in the Control group was non-significant (Z = −1.759, *p* = 0.079, RBC = −0.448). Comparisons of pre-task and post-task anxiety scores between simulated competitions were non-significant in both groups (all *p* ≥ 0.05).

**Table 2:** PANAS and state anxiety scores across simulated competitions.

Variable	Sample	Simulated Competition	Control M ± SD	Control Mdn [IQR]	Training M ± SD	Training Mdn [IQR]
PANAS positive affect	Pre	1	3.28 ± 0.49	3.36 [0.82]	3.51 ± 0.40	3.54 [0.36]
		2	3.17 ± 0.49	3.18 [0.82]	3.27 ± 0.51	3.45 [0.54]
	Post	1	3.44 ± 0.49	3.45 [0.81]	3.45 ± 0.58	3.54 [0.45]
		2	3.31 ± 0.51	3.27 [0.82]	3.36 ± 0.61	3.36 [0.91]
PANAS negative affect	Pre	1	1.69 ± 0.60	1.60 [0.60]	1.78 ± 0.63	1.70 [0.50]
		2	1.76 ± 0.51	1.60 [0.80]	1.81 ± 0.68	1.70 [1.00]
	Post	1	1.29 ± 0.20	1.30 [0.30]	1.55 ± 0.74	1.30 [0.50]
		2	1.38 ± 0.37	1.20 [0.40]	1.46 ± 0.65	1.20 [0.50]
Anxiety/STAI score	Pre	1	32.38 ± 6.51	31.00 [7.00]	34.14 ± 5.53	34.00 [7.00]
		2	33.00 ± 5.89	32.00 [8.00]	34.05 ± 7.73	33.00 [8.00]
	Post	1	30.76 ± 5.47	31.00 [5.00]	33.19 ± 8.35	31.00 [4.00]
		2	31.33 ± 6.48	31.00 [7.00]	31.29 ± 6.88	30.00 [7.00]

Note. 1 = Simulated Competition 1, pre-intervention; 2 = Simulated Competition 2, post-intervention; Pre = before the simulated competition; Post = immediately after the simulated competition; Control = group with no intervention; Training = group with SCP intervention; PANAS = Positive and Negative Affect Schedule; STAI = State-Trait Anxiety Inventory; M = mean; SD = standard deviation; Mdn = median; IQR = interquartile range. Values are presented as mean ± SD and median [IQR].

The direct contrasts compared the change in the within-task response (post-task minus pre-task) from Simulated Competition 1 to Simulated Competition 2 between the Training and Control groups. These contrasts were non-significant for PANAS positive affect (estimate = 0.18, 95% CI [−0.22, 0.58], raw *p* = 0.372, FDR-adjusted *p* = 0.990), PANAS negative affect (estimate = −0.14, 95% CI [−0.47, 0.19], raw *p* = 0.383, FDR-adjusted *p* = 0.990), and STAI state anxiety (estimate = −1.76, 95% CI [−7.82, 4.30], raw *p* = 0.559, FDR-adjusted *p* = 0.990; Table 6).

### 3.2. Self-Efficacy and Confidence

Descriptive statistics for the study-specific single-item ratings of self-efficacy, confidence, and perceived opponent self-efficacy are presented in Table 3.

**Table 3:** Self-efficacy and confidence scores across simulated competitions.

Variable	Simulated Competition	Control M ± SD	Control Mdn [IQR]	Training M ± SD	Training Mdn [IQR]
Self-efficacy	1	8.03 ± 1.39	8.00 [2.30]	8.14 ± 0.92	8.00 [1.70]
	2	7.29 ± 1.46	7.60 [2.30]	7.58 ± 2.25	8.00 [3.30]
Opponent perceived self-efficacy	1	8.30 ± 1.99	8.60 [1.30]	7.46 ± 1.32	7.00 [2.00]
	2	7.79 ± 1.42	8.30 [1.40]	7.20 ± 2.12	7.60 [2.00]
Confidence	1	5.62 ± 1.13	5.75 [1.25]	5.54 ± 1.06	5.50 [1.75]
	2	5.73 ± 0.99	5.50 [1.50]	5.51 ± 1.36	5.50 [2.00]

Note. 1 = Simulated Competition 1, pre-intervention; 2 = Simulated Competition 2, post-intervention; Control = group with no intervention; Training = group with SCP intervention; M = mean; SD = standard deviation; Mdn = median; IQR = interquartile range. Values are presented as mean ± SD and median [IQR].

Mean self-efficacy ratings decreased from 8.14 to 7.58 in the Training group and from 8.03 to 7.29 in the Control group between Simulated Competition 1 and Simulated Competition 2. The within-group change was non-significant in the Training group (*p* = 0.321; RBC = −0.258) and significant in the Control group (*p* = 0.019; RBC = −0.595). The direct between-group change contrast was non-significant (estimate = 0.18, 95% CI [−0.81, 1.17], raw *p* = 0.714, FDR-adjusted *p* = 0.990; Table 6).

Confidence ratings showed no significant change between simulated competitions in either the Training group (*p* = 0.958; RBC = −0.015) or the Control group (*p* = 0.756; RBC = 0.085). Between-group comparisons were non-significant at Simulated Competition 1 (*p* = 0.806; RBC = −0.04) and Simulated Competition 2 (*p* = 0.563; RBC = −0.09). The direct between-group change contrast was also non-significant (estimate = −0.13, 95% CI [−0.83, 0.57], raw *p* = 0.707, FDR-adjusted *p* = 0.990; Table 6).

Perceived opponent self-efficacy ratings showed no significant change between simulated competitions in the Training group (*p* = 0.701; RBC = −0.100) or the Control group (*p* = 0.054; RBC = −0.490). The between-group difference was significant at Simulated Competition 1 (*p* = 0.011; RBC = −0.46) but non-significant at Simulated Competition 2 (*p* = 0.331; RBC = −0.18). The direct between-group change contrast was non-significant (estimate = 0.26, 95% CI [−0.84, 1.35], raw *p* = 0.637, FDR-adjusted *p* = 0.990; Table 6).

### 3.3. Task Evaluation

Descriptive statistics for the five task-evaluation ratings are presented in Table 4. Comparisons between Simulated Competition 1 and Simulated Competition 2 showed no significant within-group changes in frustration (Training: *p* = 0.452, RBC = 0.20; Control: *p* = 0.943, RBC = −0.03). Perceived stress decreased non-significantly in the Training group (*p* = 0.055; RBC = −0.495), and the corresponding comparison in the Control group was also non-significant (*p* = 0.567; RBC = −0.167). Changes in perceived difficulty were non-significant in both groups (Training: *p* = 0.657, RBC = −0.13; Control: *p* = 0.107, RBC = 0.48). Effort and belief/expectancy of success showed no significant within-group changes or between-group differences at either simulated competition (all *p* ≥ 0.05). Between-group comparisons for frustration were also non-significant at both simulated competitions (all *p* ≥ 0.05).

**Table 4:** Task evaluation scores across simulated competitions.

Variable	Simulated Competition	Control M ± SD	Control Mdn [IQR]	Training M ± SD	Training Mdn [IQR]
Task evaluation effort	1	5.95 ± 2.29	7.00 [3.00]	5.62 ± 2.20	6.00 [5.00]
	2	5.76 ± 2.36	7.00 [5.00]	5.67 ± 2.13	6.00 [2.00]
Task evaluation frustration	1	3.48 ± 2.75	2.00 [4.00]	3.95 ± 2.75	3.00 [3.00]
	2	3.38 ± 1.75	3.00 [1.00]	4.38 ± 2.67	4.00 [4.00]
Task evaluation belief	1	6.62 ± 2.33	7.00 [3.00]	6.57 ± 2.56	7.00 [3.00]
	2	6.33 ± 2.35	6.00 [3.00]	5.95 ± 2.42	6.00 [4.00]
Task evaluation stress	1	3.52 ± 2.18	3.00 [3.00]	4.62 ± 1.99	5.00 [3.00]
	2	3.19 ± 2.44	3.00 [3.00]	3.62 ± 2.18	3.00 [3.00]
Task evaluation difficulty	1	4.95 ± 2.46	5.00 [4.00]	5.19 ± 1.81	5.00 [4.00]
	2	5.52 ± 2.20	6.00 [2.00]	5.05 ± 2.13	5.00 [4.00]

Note. 1 = Simulated Competition 1, pre-intervention; 2 = Simulated Competition 2, post-intervention; Control = group with no intervention; Training = group with SCP intervention; M = mean; SD = standard deviation; Mdn = median; IQR = interquartile range. Values are presented as mean ± SD and median [IQR].

The direct between-group change contrasts, calculated as the mean change from Simulated Competition 1 to Simulated Competition 2 in the Training group minus the corresponding change in the Control group, were non-significant for all five ratings: effort (estimate = 0.24, 95% CI [−1.37, 1.85], raw *p* = 0.767, FDR-adjusted *p* = 0.990), frustration (estimate = 0.52, 95% CI [−1.28, 2.33], raw *p* = 0.561, FDR-adjusted *p* = 0.990), belief/expectancy of success (estimate = −0.33, 95% CI [−2.21, 1.55], raw *p* = 0.722, FDR-adjusted *p* = 0.990), perceived stress (estimate = −0.67, 95% CI [−2.13, 0.80], raw *p* = 0.363, FDR-adjusted *p* = 0.990), and perceived difficulty (estimate = −0.71, 95% CI [−1.93, 0.50], raw *p* = 0.242, FDR-adjusted *p* = 0.990; Table 6).

### 3.4. Causal Attributions

Descriptive statistics for the five causal-attribution ratings and the internal and external attribution indices are presented in Table 5. Luck attribution increased from Simulated Competition 1 to Simulated Competition 2 in the Training group (*p* = 0.007; RBC = 1.00), with nine participants reporting higher scores, none reporting lower scores, and 12 reporting unchanged scores. The corresponding increase in the Control group was non-significant (*p* = 0.087; RBC = 0.69). Ability/capacity, effort, task difficulty, and opponent skill attributions showed no significant changes between simulated competitions in either group, and between-group comparisons at each simulated competition were non-significant for these four ratings (all *p* ≥ 0.05).

**Table 5:** Causal attribution scores across simulated competitions.

Variable	Simulated Competition	Control M ± SD	Control Mdn [IQR]	Training M ± SD	Training Mdn [IQR]
Attribution capacity	1	4.05 ± 1.12	4.00 [2.00]	4.43 ± 0.87	5.00 [1.00]
	2	4.38 ± 0.97	5.00 [1.00]	4.38 ± 0.86	5.00 [1.00]
Attribution luck	1	1.57 ± 0.87	1.00 [1.00]	1.29 ± 0.64	1.00 [0.00]
	2	2.05 ± 1.07	2.00 [2.00]	2.00 ± 0.95	2.00 [2.00]
Attribution effort	1	4.29 ± 0.85	5.00 [1.00]	4.00 ± 1.05	4.00 [2.00]
	2	4.00 ± 0.95	4.00 [2.00]	3.76 ± 0.89	3.00 [2.00]
Attribution difficulty	1	3.38 ± 1.47	3.00 [2.00]	3.14 ± 1.11	3.00 [1.00]
	2	3.19 ± 0.98	3.00 [1.00]	2.76 ± 0.94	3.00 [0.00]
Attribution opponent skills	1	2.33 ± 1.43	3.00 [2.00]	1.81 ± 1.25	1.00 [2.00]
	2	2.05 ± 1.50	1.00 [2.00]	1.52 ± 1.08	1.00 [0.00]
Internal attribution index	1	4.17 ± 0.75	4.50 [1.50]	4.21 ± 0.80	4.00 [1.50]
	2	4.19 ± 0.84	4.50 [1.00]	4.07 ± 0.68	4.00 [1.00]
External attribution index	1	2.43 ± 0.78	2.33 [1.00]	2.08 ± 0.56	2.33 [0.67]
	2	2.43 ± 0.82	2.33 [1.33]	2.10 ± 0.67	1.67 [0.67]

Note. 1 = Simulated Competition 1, pre-intervention; 2 = Simulated Competition 2, post-intervention; Control = group with no intervention; Training = group with SCP intervention; M = mean; SD = standard deviation; Mdn = median; IQR = interquartile range. Values are presented as mean ± SD and median [IQR].

The direct between-group change contrasts, calculated as the mean change from Simulated Competition 1 to Simulated Competition 2 in the Training group minus the corresponding change in the Control group, were non-significant for all five ratings: ability/capacity (estimate = −0.38, 95% CI [−1.12, 0.36], raw *p* = 0.303, FDR-adjusted *p* = 0.990), luck (estimate = 0.24, 95% CI [−0.38, 0.86], raw *p* = 0.442, FDR-adjusted *p* = 0.990), effort (estimate = 0.05, 95% CI [−0.67, 0.76], raw *p* = 0.893, FDR-adjusted *p* = 0.990), task difficulty (estimate = −0.19, 95% CI [−1.00, 0.62], raw *p* = 0.639, FDR-adjusted *p* = 0.990), and opponent skill (estimate = 0.00, 95% CI [−0.93, 0.93], raw *p* = 1.000, FDR-adjusted *p* = 1.000; Table 6).

The internal attribution index, calculated as the mean of ability/capacity and effort, showed no significant change between simulated competitions in either group. In the Training group, scores were M = 4.21, SD = 0.80 at Simulated Competition 1 and M = 4.07, SD = 0.68 at Simulated Competition 2 (*p* = 0.469; RBC = −0.273). Corresponding scores in the Control group were M = 4.17, SD = 0.75 and M = 4.19, SD = 0.84 (*p* = 0.843; RBC = 0.077). The direct between-group change contrast was non-significant (estimate = −0.17, 95% CI [−0.72, 0.38], raw *p* = 0.542, FDR-adjusted *p* = 0.990; Table 6).

The external attribution index, calculated as the mean of luck, task difficulty, and opponent skill, also showed no significant change between simulated competitions in either group. In the Training group, scores were M = 2.08, SD = 0.56 at Simulated Competition 1 and M = 2.10, SD = 0.67 at Simulated Competition 2 (*p* = 1.000; RBC = 0.010). Corresponding scores in the Control group were M = 2.43, SD = 0.78 and M = 2.43, SD = 0.82 (*p* = 0.814; RBC = −0.090). The direct between-group change contrast was non-significant (estimate = 0.02, 95% CI [−0.45, 0.48], raw *p* = 0.945, FDR-adjusted *p* = 0.990; Table 6).

**Table 6:** Direct Training-versus-Control contrasts for psychological outcomes.

Outcome	Contrast	Estimate	95% CI	Raw *p*	FDR-Adjusted *p*
SAM—Valence	A †	−0.05	[−0.54, 0.44]	0.846	0.990
SAM—Activation	A †	0.14	[−0.42, 0.71]	0.612	0.990
SAM—Dominance/control	A †	−0.14	[−0.59, 0.30]	0.518	0.990
SAM arithmetic sum ‡	A †	−0.05	[−1.03, 0.93]	0.922	0.990
PANAS—Positive affect	B	0.18	[−0.22, 0.58]	0.372	0.990
PANAS—Negative affect	B	−0.14	[−0.47, 0.19]	0.383	0.990
STAI—State anxiety	B	−1.76	[−7.82, 4.30]	0.559	0.990
Self-efficacy	A	0.18	[−0.81, 1.17]	0.714	0.990
Confidence	A	−0.13	[−0.83, 0.57]	0.707	0.990
Opponent perceived self-efficacy	A	0.26	[−0.84, 1.35]	0.637	0.990
Task evaluation—Effort	A	0.24	[−1.37, 1.85]	0.767	0.990
Task evaluation—Frustration	A	0.52	[−1.28, 2.33]	0.561	0.990
Task evaluation—Belief	A	−0.33	[−2.21, 1.55]	0.722	0.990
Task evaluation—Stress	A	−0.67	[−2.13, 0.80]	0.363	0.990
Task evaluation—Difficulty	A	−0.71	[−1.93, 0.50]	0.242	0.990
Attribution—Ability	A	−0.38	[−1.12, 0.36]	0.303	0.990
Attribution—Luck	A	0.24	[−0.38, 0.86]	0.442	0.990
Attribution—Effort	A	0.05	[−0.67, 0.76]	0.893	0.990
Attribution—Task difficulty	A	−0.19	[−1.00, 0.62]	0.639	0.990
Attribution—Opponent skill	A	0.00	[−0.93, 0.93]	1.000	1.000
Internal attribution index	A	−0.17	[−0.72, 0.38]	0.542	0.990
External attribution index	A	0.02	[−0.45, 0.48]	0.945	0.990

Note. Each contrast included 21 participants per group. A = Training-minus-Control difference in mean change from Simulated Competition 1 to Simulated Competition 2. B = Training-minus-Control difference in the change across simulated competitions in the within-task response: (post-task − pre-task at Simulated Competition 2) − (post-task − pre-task at Simulated Competition 1). Estimates are in the original outcome units. Positive estimates indicate a larger increase or smaller decrease in Training; they do not necessarily indicate improvement. Confidence intervals and raw *p*-values are from Welch’s unequal-variance comparisons. Benjamini–Hochberg adjustment was applied jointly to the 22 unrounded raw *p*-values. The 95% confidence intervals were not adjusted for multiplicity. † The SAM rows reproduce the between-competition comparisons from the existing analysis dataset. The assessment phase represented by the available SAM columns requires confirmation; these estimates are not four-measurement Group × Competition × Assessment-phase contrasts. ‡ This row reproduces the arithmetic sum of the three SAM dimensions included in the previous analysis. It should not be interpreted as a validated global affect score.

## 4. Discussion

The present findings provide an exploratory, multidimensional description of psychological outcomes assessed alongside an eight-week SCP neurofeedback program in competitive golfers. Interpreted within the competition–stress framework proposed by Salvador and Costa [3], self-efficacy, perceived workload, and post-competition attribution occupy theoretically meaningful positions in the antecedent, competition, and recovery phases of that model; this framework motivated which constructs were measured, but the present psychological data do not test whether SCP training engages the neural or neuroendocrine mechanisms the model describes. The analysis reported here, using direct between-group contrasts of pre-to-post change with 95% confidence intervals and FDR correction, did not find a significant differential treatment effect for any of the psychological outcomes assessed. The discussion below therefore describes the observed within-group patterns as exploratory and hypothesis-generating, and explicitly separates the theoretical rationale from what the data can support.

Affective measures suggested a largely stable emotional tone across simulated competitions at the between-simulated competition level. SAM scores remained broadly comparable over time, and negative affect and anxiety scores did not show between-simulated competition changes. Within the Training group, state anxiety decreased from pre- to post-task at Simulated Competition 2, a pattern absent within Simulated Competition 1; as a between-group treatment effect, this is not statistically supported, since the direct Group × Simulated Competition × Task-phase contrast for anxiety was not significant, and the Control group changed in the same direction at Simulated Competition 2. Within the Salvador and Costa [3] model, post-competition anxiety reduction is one candidate marker of the active/proactive coping response, but the present data cannot establish that SCP training produced this pattern differentially; the within-group observation is offered as a hypothesis for confirmatory testing rather than as evidence of improved within-competition emotional regulation.

Self-efficacy is one of the cognitive determinants positioned in the antecedent phase of the Salvador and Costa [3] model. Descriptively, the Control group’s self-efficacy declined significantly within-group from Simulated Competition 1 to Simulated Competition 2, while the Training group’s decline did not reach within-group significance; however, the direct between-group contrast of change was not significant. A significant within-group result in one arm and a non-significant result in the other do not establish a between-group difference, so these data do not support the interpretation that SCP training had effects on self-efficacy in relation to the Control condition. Whether anticipatory cortical regulation influences the antecedent phase of the competition–stress model remains an open question for adequately powered, confirmatory designs.

Regarding subjective task demands, the study-specific task-evaluation ratings were largely stable across simulated competitions in both groups. The within-group reduction in perceived stress observed in the Training group did not reach significance, and the direct between-group contrast of change was not significant. The “effort without distress” profile described by Frankenhaeuser [4] and incorporated into the Salvador and Costa [3] model remains a plausible framework for future work with neuroendocrine measures, but the present workload ratings do not provide evidence that SCP training decoupled evaluative demand from distress.

At the level of competitive appraisal and causal interpretation, attribution patterns remained predominantly internal (effort and ability) in both groups. Luck attribution increased within the Training group from Simulated Competition 1 to Simulated Competition 2, but the direct between-group contrast of change was not significant, and a similar, non-significant within-group increase occurred in the Control group. Weiner’s [22] attribution framework offers a plausible interpretation of increased external-unstable attributions as reflecting flexible reappraisal, but this study’s checklist has not been separately validated and the present data do not establish that this pattern is specific to, or caused by, SCP training.

Taken together, the direct between-group analysis did not identify a statistically supported differential treatment effect for any of the psychological outcomes assessed, including self-efficacy, perceived stress, luck attribution, and within-task anxiety regulation. This pattern is consistent with the companion neuroendocrine and heart-rate-variability report on the same 42-participant cohort [28], where cortisol, DHEA, and HRV group-related and correlational findings likewise did not survive Benjamini–Hochberg FDR correction and were, for that reason, described by the authors as preliminary and hypothesis-generating rather than confirmatory; performance also did not improve significantly in that report. The convergence of this pattern across independently analyzed outcome domains from the same trial—psychological, neuroendocrine/autonomic, and behavioral—strengthens the case for treating the present psychological findings the same way. The competition–stress framework of Salvador and Costa [3] remains a coherent lens for generating hypotheses about how SCP-related neural self-regulation might, in principle, relate to appraisal, effort-related distress, and post-competition reappraisal, but the present exploratory data neither confirm nor rule out these pathways. The within-group patterns described above are hypothesis-generating and should motivate, rather than substitute for, adequately powered confirmatory trials that incorporate an active comparator and neuroendocrine measures (cortisol, testosterone) to test the full model directly.

### Limitations and Future Directions

Several limitations should be considered when interpreting the present findings. First, and most importantly, this is an exploratory trial and the results should be treated as hypothesis-generating: the direct between-group analysis did not establish a significant intervention-by-time effect for any of the psychological outcomes assessed, and the within-group patterns reported for anxiety, self-efficacy, and luck attribution should not be read as demonstrated treatment effects. Second, the very large number of outcomes and timepoints creates substantial multiplicity; although the present reanalysis applies an FDR correction across the full family of direct treatment contrasts, the within-group tests reported descriptively in Table 1, Table 2, Table 3, Table 4 and Table 5 were not corrected and carry an elevated risk of false-positive findings. Third, the sample size (*n* = 42; *n* = 21 per group), while consistent with comparable pilot RCTs in the neurofeedback and sport psychology literature, is underpowered for the small-to-moderate effect sizes observed in the direct contrasts and does not support conclusions of equivalence or absence of an effect. Fourth, the absence of an active control condition (e.g., sham neurofeedback or an alternative cognitive training protocol) means that expectancy effects, non-specific attention, and therapeutic alliance cannot be disentangled from protocol-specific SCP training effects; while outcome assessors were blinded to group assignment throughout the trial, participants could not be blinded given the wait-list design, which still permits differential expectancy effects between groups. Fifth, the reliance on self-report instruments introduces demand characteristics and response biases that are difficult to exclude in open-label designs, and several instruments (the study-specific task-evaluation ratings and the sport-adapted attribution checklist) have not been independently validated. Sixth, perceived opponent self-efficacy differed between groups at baseline (*p* = 0.011), a baseline imbalance that should be reported alongside the outcome analyses. Seventh, generalizability is restricted to experienced competitive golfers in putting contexts, and extension to other precision sports, team sports, or different competitive formats requires caution. Finally, the exact handicap strata and cut-offs used for stratification during randomization could not be recovered, preventing independent reproduction of this component of the randomization procedure.

Future confirmatory studies should incorporate: (a) larger, prospectively powered samples based on the modest effect sizes and wide confidence intervals observed in the present direct contrasts; (b) pre-registration of a limited set of primary outcomes, with all remaining outcomes explicitly labeled exploratory; (c) an active sham-neurofeedback control with documented allocation concealment and masking; (d) neuroendocrine biomarkers (cortisol, testosterone) aligned with the competition–stress model; and (e) longer follow-up periods to assess the durability of any effects beyond the immediate post-training simulated competition. The present study provides an empirical and theoretical foundation for designing and powering such a trial, without itself providing convincing evidence of psychological benefit attributable to SCP neurofeedback.

## 5. Conclusions

This study analysed the psychological outcomes following an eight-week SCP neurofeedback intervention. None of these direct contrasts reached statistical significance, including self-efficacy, perceived stress, luck attribution, and within-task anxiety regulation. The reported analyses therefore do not provide evidence that SCP neurofeedback improved the psychological outcomes assessed relative to a wait-list control. Within-group changes in anxiety, self-efficacy, and luck attribution observed in one or both groups are exploratory, hypothesis-generating observations and should not be interpreted as treatment effects or as evidence of a psychological or neural mechanism. The small sample and the width of the confidence intervals also mean that this study cannot establish equivalence between groups or rule out clinically or practically relevant effects. Future trials should use prespecified primary outcomes, a treatment-effect model appropriate to the repeated-measures design, adequately powered samples, and a credible active comparator before drawing conclusions about the psychological benefits of SCP neurofeedback in competitive sport.

## Data Availability

The datasets generated and/or analyzed during the current study are not publicly available due to participant confidentiality and data protection considerations. The sample consists of competitive athletes, and some of the collected information could potentially facilitate identification when combined with individual demographic, psychological, or performance-related characteristics. However, appropriately anonymized data may be made available by the corresponding author upon reasonable request and subject to relevant ethical and institutional requirements.

## References

[1] Strehl U., Aggensteiner P., Wachtlin D., Brandeis D., Albrecht B., Arana M., Bach C., Banaschewski T., Bogen T., Flaig-Röhr A. (2017). Neurofeedback of slow cortical potentials in children with attention-deficit/hyperactivity disorder: A multicenter randomized trial controlling for unspecific effects. Front. Hum. Neurosci..

[2] Lizama E., Lorenzon L., Pereira C., Serrano M.A. (2025). Enhancing neural efficiency in competitive golfers: Effects of slow cortical potential neurofeedback on modulation of beta activity—An exploratory randomized controlled trial. NeuroSci.

[3] Salvador A., Costa R. (2009). Coping with competition: Neuroendocrine responses and cognitive variables. Neurosci. Biobehav. Rev..

[4] Frankenhaeuser M., Appley M.H., Trumbull R. (1986). A psychobiological framework for research on human stress and coping. Dynamic of Stress: Physiological, Psychological, and Social Perspectives.

[5] Friedman N.P., Robbins T.W. (2021). The role of prefrontal cortex in cognitive control and executive function. Neuropsychopharmacology.

[6] Arnsten A.F., Joyce M.K.P., Roberts A.C. (2022). The aversive lens: Stress effects on the prefrontal–cingulate cortical pathways that regulate emotion. Neurosci. Biobehav. Rev..

[7] Palamarchuk I.S., Vaillancourt T. (2021). Mental resilience and coping with stress: A comprehensive, multi-level model of cognitive processing, decision making, and behavior. Front. Behav. Neurosci..

[8] Dehais F., Lafont A., Roy R., Fairclough S. (2020). A neuroergonomics approach to mental workload, engagement and human performance. Front. Neurosci..

[9] Causse M., Lepron E., Mandrick K., Peysakhovich V., Berry I., Callan D., Rémy F. (2021). Facing successfully high mental workload and stressors: An fMRI study. Hum. Brain Mapp..

[10] Lundkvist E., Gustafsson H., Björklund G., Davis P., Ivarsson A. (2021). Relating competitive golfers’ perceived emotions and performance. Percept. Mot. Ski..

[11] Hill D., Barker J., Steptoe K. (2023). The Psychology of Golf Performance Under Pressure.

[12] Avery C., Shipherd A.M., Gomez S., Barczarenner K. (2022). Exploring stress mindset and perceived stress between college student-athletes and non-athletes. Int. J. Exerc. Sci..

[13] Meijen C., Turner M., Jones M.V., Sheffield D., McCarthy P. (2020). A theory of challenge and threat states in athletes: A revised conceptualization. Front. Psychol..

[14] Shin M., Kim Y., Park S. (2019). Effects of state anxiety and ego depletion on performance change in golf putting: A hierarchical linear model application. Percept. Mot. Ski..

[15] Martins L.A., Schiavo A., Paz L.V., Xavier L.L., Mestriner R.G. (2024). Neural underpinnings of fine motor skills under stress and anxiety: A review. Physiol. Behav..

[16] Bradley M.M., Lang P.J. (1994). Measuring emotion: The self-assessment manikin and the semantic differential. J. Behav. Ther. Exp. Psychiatry.

[17] Watson D., Clark L.A., Tellegen A. (1988). Development and validation of brief measures of positive and negative affect: The PANAS scales. J. Personal. Soc. Psychol..

[18] Bandura A. (2000). Self-Efficacy.

[19] López-Rodríguez L., Carballido L.G.G., Montoya-Romero C.A., Suárez-Rodríguez M.C., González-Rabeiro M., Charlot-Cardoza O., Yañez-Rivera A., Feria-Madueño A. (2025). Self-efficacy in high-performance sports: A systematic review and meta-analysis. Psychol. Russ. State Art..

[20] Lochbaum M., Sisneros C., Cooper S., Terry P.C. (2023). Pre-event self-efficacy and sports performance: A systematic review with meta-analysis. Sports.

[21] Hertzum M. (2021). Reference values and subscale patterns for the task load index (TLX): A meta-analytic review. Ergonomics.

[22] Weiner B. (1985). An attributional theory of achievement motivation and emotion. Psychol. Rev..

[23] Brun L., Pansu P., Dompnier B. (2021). The role of causal attributions in determining behavioral consequences: A meta-analysis from an intrapersonal attributional perspective in achievement contexts. Psychol. Bull..

[24] Chamberlain S.T., Hale B.D. (2007). Competitive state anxiety and self-confidence: Intensity and direction as relative predictors of performance on a golf putting task. Anxiety Stress Coping.

[25] Smith R., Friston K.J., Whyte C.J. (2022). A step-by-step tutorial on active inference and its application to empirical data. J. Math. Psychol..

[26] Friston K. (2018). Does predictive coding have a future?. Nat. Neurosci..

[27] Ursin H., Eriksen H.R. (2004). The cognitive activation theory of stress. Psychoneuroendocrinology.

[28] Lizama E., Carcamo-Herrera B., Lopez G., Pereira C., Serrano M.A. (2026). Modulation of neuroendocrine and autonomic responses to competitive stress by slow cortical potentials training in golfers: An exploratory randomized controlled trial. Psychoneuroendocrinology.

[29] Dufey M., Fernández A.M. (2012). Validez y confiabilidad del Positive Affect and Negative Affect Schedule (PANAS) en estudiantes universitarios chilenos. Rev. Iberoam. Diagnóstico Eval.-E Aval. Psicológica.

[30] Spielberger C.D., Gorsuch R.L., Lushene R., Vagg P.R., Jacobs G.A. (1983). Manual for the State-Trait Anxiety Inventory.

[31] Vera-Villarroel P., Celis-Atenas K., Córdova-Rubio N., Buela-Casal G., Spielberger C.D. (2007). Análisis preliminar y datos normativos del Cuestionario de Ansiedad Estado-Rasgo en adolescentes y adultos de la ciudad de Santiago de Chile. Ter. Psicológica.

[32] Myers N.D., Feltz D.L., Tenenbaum G., Eklund R.C. (2007). From self-efficacy to collective efficacy in sport: Transitional methodological issues. Handbook of Sport Psychology.

[33] Costa R., Salvador A. (2012). Associations between success and failure in a face-to-face competition and psychobiological parameters in young women. Psychoneuroendocrinology.

[34] Baggett H.L., Saab P.G., Carver C.S. (1996). Appraisal, coping, task performance, and cardiovascular responses during the evaluated speaking task. Personal. Soc. Psychol. Bull..

[35] Carrillo E., Moya-Albiol L., González-Bono E., Salvador A., Ricarte J., Gómez-Amor J. (2001). Gender differences in cardiovascular and electrodermal responses to public speaking task: The role of anxiety and mood states. Int. J. Psychophysiol..

[36] Prosoli R., Banai B., Renata R., Lochbaum M., Cooper S., Jelić M. (2021). Causal attributions for success and failure among athletes: Validation of the Croatian version of the revised Causal Dimension Scale (CDS-II). Pedagog. Phys. Cult. Sports.

[37] Dias G., Couceiro M., Mendes P., Gomes R., Mendes R., Vaz V., Martins F., Gama J., Castro M.A. (2021). Golf-Putting Performance in Skilled Golfers at Different Distances to the Hole. Appl. Sci..

